# Random plasma glucose in early pregnancy is a better predictor of gestational diabetes diagnosis than maternal obesity

**DOI:** 10.1007/s00125-015-3811-5

**Published:** 2015-11-20

**Authors:** Claire L. Meek, Helen R. Murphy, David Simmons

**Affiliations:** The Wellcome Trust–MRC Institute of Metabolic Science, Metabolic Research Laboratories, University of Cambridge, Addenbrooke’s Hospital, Box 289, Hills Road, Cambridge, CB2 0QQ UK; Wolfson Diabetes and Endocrinology Clinic, Cambridge University Hospitals, Addenbrooke’s Hospital, Cambridge, UK; Department of Clinical Biochemistry, Cambridge University Hospitals, Addenbrooke’s Hospital, Cambridge, UK; Department of Medicine, University of East Anglia, Norwich Medical School, Norwich, UK; School of Medicine, University of Western Sydney, Campbelltown, NSW Australia

## Abstract

**Aims/hypothesis:**

Asymptomatic pregnant women are screened for gestational diabetes (GDM) at 24–28 weeks’ gestation. Recent guidelines also recommend screening early in gestation to identify undiagnosed pre-existing overt diabetes. We assessed the performance of random plasma glucose (RPG) testing at antenatal booking in predicting GDM diagnosis later in pregnancy.

**Methods:**

Data from 25,543 consecutive singleton pregnancies at the Rosie Hospital in Cambridge (UK) were obtained from hospital electronic records as a service evaluation. All women were invited for an antenatal RPG (12–16 weeks) and a 50 g glucose challenge test (GCT; 24–28 weeks) with a 75 g OGTT if GCT >7.7 mmol/l (139 mg/dl).

**Results:**

At booking, 17,736 women had an RPG that was able to predict GDM (receiver operating characteristic AUC 0.8) according to various diagnostic criteria in common use. A cut-off point of ≥7.5 mmol/l (135 mg/dl) gave a sensitivity of 0.70 and a specificity of 0.90 for GDM diagnosis. Theoretically, using this screening policy, 13.2% of women would have been categorised at high risk (26.3% had GDM) and 86.8% of women at low risk (1.7% had GDM). RPG performed better than maternal age (AUC 0.60) or BMI (AUC 0.65) at predicting GDM diagnosis.

**Conclusions/interpretation:**

RPG at booking has reasonable performance as a screening test and is better than maternal age or BMI for identifying women at high risk of GDM. RPG cannot replace OGTT for diagnosis but it may be useful to exclude women who do not need further investigation for GDM and to identify women who could be prioritised for early diagnosis or lifestyle interventions.

**Electronic supplementary material:**

The online version of this article (doi:10.1007/s00125-015-3811-5) contains peer-reviewed but unedited supplementary material, which is available to authorised users.

## Introduction

Gestational diabetes (GDM), defined as carbohydrate intolerance causing hyperglycaemia with first onset or recognition during pregnancy, is associated with adverse maternal and fetal outcomes [1, 2]. The Hyperglycaemia and Adverse Perinatal Outcomes (HAPO) study identified that all degrees of hyperglycaemia are linked linearly to adverse outcomes in pregnancy, with no obvious inflection point for this risk [3]. This has led to considerable difficulty in defining GDM. The International Association of the Diabetes in Pregnancy Study Groups (IADPSG) recommended setting diagnostic cut-off points at a level consistent with an OR of 1.75 [4] (see electronic supplementary material [ESM] Table 1), which has resulted in a larger number of diagnoses. These criteria have been adopted by the WHO [5] and the ADA [6] but have not been universally accepted [7, 8], in part due to concerns about resource allocation with the increasing prevalence of GDM and concerns about excessive medicalisation of healthy pregnancy [9]. Although many countries are adopting the WHO 2013 criteria, there remains great heterogeneity of diagnostic criteria used for GDM, even within the same country [10].

There is also considerable controversy about how best to identify women with GDM. The ADA and US Preventive Services Task Force (USPSTF) recommend that all pregnant women should be screened at 24–28 weeks unless they are known to have pre-existing diabetes [6]. The American Congress of Obstetricians and Gynecologists (ACOG) guidelines agree that all women should be screened at 24–28 weeks’ gestation but suggest that this could be performed by assessment of ‘the patient’s medical history, clinical risk factors, or laboratory screening test results to determine blood glucose levels’ [11]. Guidelines published by the National Institute for Health and Care Excellence (NICE) in the UK recommend screening only women with risk factors, including obesity, previous GDM, family history of diabetes or ethnicity with a high diabetes prevalence [8]. Although universal screening policies whereby all women are screened biochemically for GDM are considered expensive, there is some concern that risk-factor-based approaches miss many cases who might otherwise benefit from treatment [12] and create added complexity for healthcare professionals conducting the screening [13].

There is also considerable controversy regarding the type and timing of blood tests with which to diagnose GDM. The ACOG and USPSTF recommendations from the USA have favoured a two-step approach using a 50 g glucose challenge test (GCT) and a confirmatory test using the 100 g OGTT [11]. The WHO, ADA and IADPSG all recommend a one-step approach, using a 75 g OGTT with glucose determination carried out at baseline and at 1 and 2 h after the glucose load. The NICE guidelines recommend a 75 g OGTT with glucose measurement at baseline and at 2 h post load. Other groups have suggested that tests such as fasting plasma glucose [14] or random plasma glucose (RPG) [15] might have validity in screening for GDM, either alone or as a method of rationing OGTTs. A previous systematic review of the use of RPG to screen for GDM concluded that there was inadequate evidence to support the use of RPG, but only six relevant studies (including a total of 3,537 women) were identified [16].

In our institution, an RPG is taken at antenatal booking (12–16 weeks) to exclude overt diabetes [17]. Because women who develop GDM have abnormal glucose handling or insulin resistance prior to pregnancy, we hypothesised that the RPG may also be able to identify women who may later develop GDM. The aim of this study was to assess the usefulness of an RPG taken at antenatal booking as a screening test for GDM, diagnosed at any point during pregnancy.

## Methods

### Population and standard care

As described previously [18], data from all singleton pregnancies (2004–2008) at the Rosie Hospital, Cambridge Universities NHS Foundation Trust were obtained retrospectively from hospital medical and obstetric records as part of an approved service evaluation. At that time in our institution, all pregnant women had been invited to attend an antenatal appointment at which RPG (*n* = 17,736; typically 12–16 weeks’ gestation) was measured. Women with RPG >7.0 mmol/l or who had a previous diagnosis of GDM were offered an early 75 g OGTT. All women without known GDM/pre-existing diabetes were screened at 24–28 weeks with a 50 g GCT: women with a GCT result >7.7 mmol/l (139 mg/dl) were then referred for a 75 g OGTT [18]. Additional OGTTs were performed in later pregnancy if symptoms were present. Therefore, all women who had an OGTT (*n* = 3,848) had already had at least one abnormal glucose test result during pregnancy, symptoms consistent with hyperglycaemia or GDM in a previous pregnancy. Women with known pre-existing diabetes were excluded from the study. The study period of 2004–2008 was chosen as electronic records, screening procedures and treatment protocols were constant during this time.

### Laboratory analysis

Both venous and capillary blood samples were used during 2004–2008 for glucose testing in our institution. Venous blood was collected using fluoride–oxalate tubes and analysed using a hexokinase method (Dimension RXL MAX Clinical Chemistry System; Siemens Healthcare Diagnostics, Deerfield, IL, USA) in our accredited laboratory (Clinical Pathology Accreditation, UK Accreditation Service, Feltham, UK). Capillary samples were analysed using the Bayer Elite glucose monitoring system (Bayer, Newbury, UK). Although both laboratory and point-of-care methods were regularly calibrated, small differences exist between capillary and venous glucose testing [19]. The same diagnostic criteria were used for both capillary and venous tests.

### Statistical analysis

Receiver operating characteristic (ROC) curves were used to estimate AUC and the 95% CI. Statistical analysis was performed using STATA (version 12.0; StataCorp, College Station, TX, USA).

## Results

Records were obtained for 25,789 births; 25,543 records were included in the analysis after exclusion of pregnancies resulting in miscarriage (*n* = 59) or termination (*n* = 65) and records with no birthweight information (*n* = 3), duplicate data (*n* = 20) and data consistent with overt diabetes (RPG ≥11.1 mmol/l at booking; *n* = 99). Of these, only 17,736 pregnancies had a documented RPG at booking. Those without RPG measurements recorded have been described more thoroughly elsewhere [17].

Baseline characteristics of women are described according to the presence or absence of GDM according to the IADPSG criteria (Table 1). As expected, women with GDM present had higher rates of obesity (BMI ≥30 kg/m^2^), higher age at delivery and were more likely to give birth to a macrosomic or large-for-gestational-age (LGA) infant compared with women who did not have GDM.

**Table 1:** Characteristics of all pregnancies and those identified as GDM-positive and GDM-negative according to the IADPSG criteria

Characteristic	All pregnancies	GDM-negative (IADPSG)	GDM-positive (IADPSG)
No. of pregnancies	25,543	24,362	1,181
Maternal age ≥30 years at delivery	15,773 (61.8)	14,890 (61.1)	883 (74.8)
Maternal smoking at booking	2,416 (9.5)	2,342 (9.6)	74 (6.3)
Maternal white ethnicity	22,762 (89.3)	21,785 (89.6)	977 (82.8)
Maternal obesity	3,016 (13.9)	2,701 (13.1)	315 (30.0)
Primiparous	9,895 (38.8)	9,437 (38.8)	458 (38.9)
Macrosomia (BW >4 kg)	3,097 (12.1)	2,854 (11.7)	243 (20.6)
LGA (BW >90th percentile)	3,010 (12.2)	2,700 (11.5)	310 (26.9)
Method of delivery			
SVD	15,321 (60.0)	14,790 (60.7)	531 (45.0)
CS	6,795 (26.6)	6,301 (25.9)	494 (41.8)

Data are shown as *n* (%)

Note that approximately 99.9% of records had data available for pregnancy outcome, mode of delivery and antenatal complications but only 84.9% of records had data available for their usual maternal adult BMI

BW, birthweight; CS, Caesarean section; SVD, spontaneous vertex delivery

**Fig. 1:**
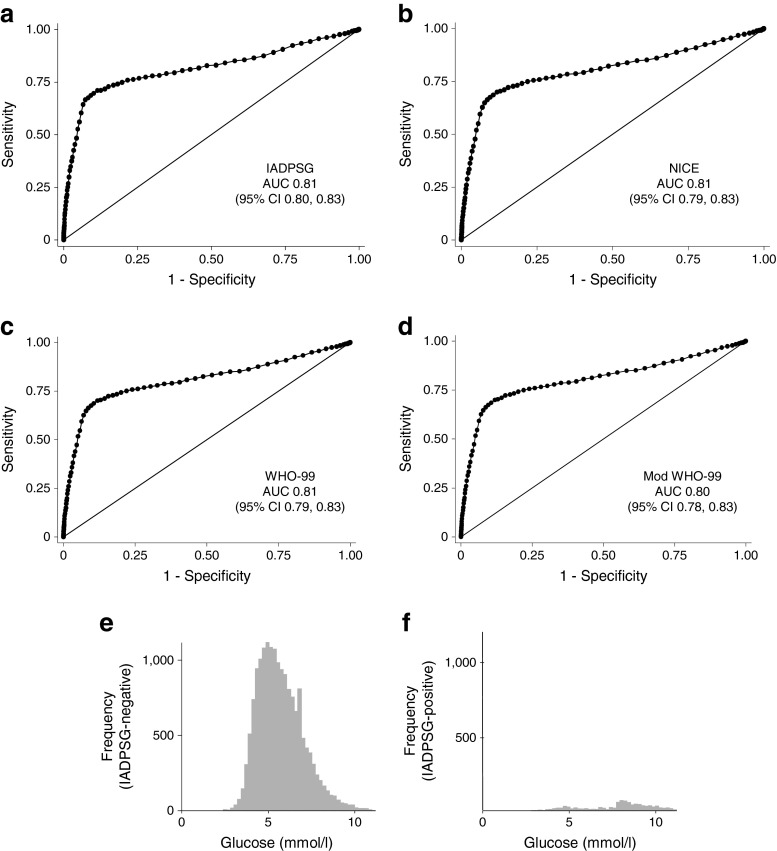
(**a**–**d**) ROC curves for the use of RPG to predict GDM diagnosed using the IADPSG criteria (**a**), the NICE 2015 criteria (**b**), the WHO 1999 (WHO-99) criteria (**c**) and the modified (Mod) WHO 1999 criteria (**d**); AUC and 95% CIs are also shown. The distribution of RPG values in women who were negative (**e**) and positive (**f**) for GDM according to the IADPSG criteria

The ability of the RPG to predict GDM was tested using ROC curves (Fig. 1). RPG was able to predict GDM according to the IADPSG (*n* = 1,181 positive diagnoses, *n* = 884 with RPG; AUC 0.81; 95% CI 0.80, 0.83), NICE 2015 (*n* = 1,055 positive diagnoses, *n* = 806 with RPG; AUC 0.81; 95% CI 0.79, 0.83), WHO 1999 (*n* = 1,016 positive diagnoses; *n* = 775 with RPG; AUC 0.81; 95% CI 0.79, 0.83) and Modified WHO 1999 (*n* = 1,025 positive diagnoses; *n* = 782 with RPG; AUC 0.80; 95% CI 0.78, 0.83) criteria.

Using a cut-off value of RPG ≥7.5 mmol/l (135 mg/dl), which produced best overall performance of sensitivity and specificity, RPG was able to predict GDM diagnosis using IADPSG (sensitivity 0.70, specificity 0.90), NICE 2015 (sensitivity 0.69, specificity 0.89), WHO 1999 (sensitivity 0.69, specificity 0.89) and Modified WHO 1999 (sensitivity 0.69, specificity 0.89) criteria. In this dataset of 17,736 pregnancies with RPG data, 15,396 women (86.81%) fell below this threshold and 2,340 fell above the threshold (13.19%).

As the clinical value of RPG would be in excluding women who do not need further investigation for GDM, a higher cut-off value of ≥8.5 mmol/l (153 mg/dl) was also assessed to maximise specificity while providing acceptable sensitivity. At this level, RPG was able to predict GDM according to IADPSG (sensitivity 0.43, specificity 0.97), NICE 2015 (sensitivity 0.42, specificity 0.96), WHO 1999 (sensitivity 0.42, specificity 0.96) and Modified WHO 1999 (sensitivity 0.42, specificity 0.96) criteria. In this dataset of 17,736 pregnancies with recorded RPG, 16,789 women fell below this threshold and 947 fell above the threshold.

As the range of RPG values was considerable in women who later developed GDM (see Fig. 1e), a cut-off point of around ≥4.7 mmol/l (85 mg/dl) was required to give a sensitivity of 90% using any diagnostic criteria. Of the 3,863 women who had values <4.7 mmol/l, 68 (1.76%) were eventually diagnosed with GDM according to IADPSG criteria.

**Fig. 2:**
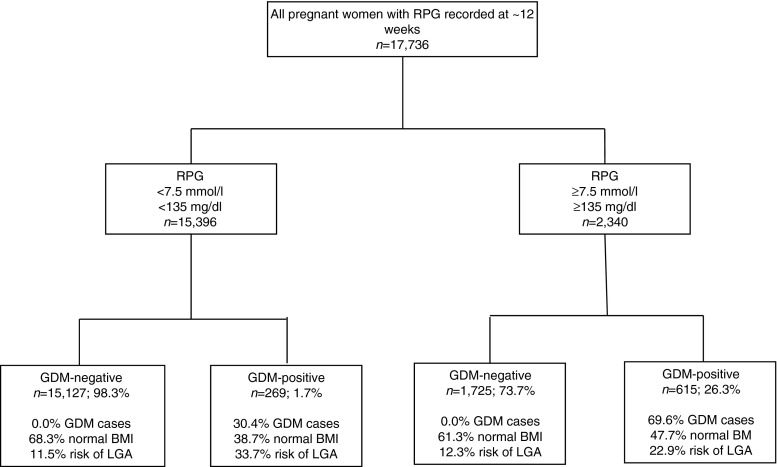
Features of women with RPG above and below the 7.5 mmol/l (135 mg/dl) cut-off point

Theoretically, adopting an RPG screening policy in this population using IADPSG criteria with a cut-off point of ≥7.5 mmol/l (135 mg/dl) would have identified 2,340 women as being at high risk of GDM (Fig. 2; 615 [26.3%] were later found to be positive for GDM). This screening policy would also have identified 15,396 women as being at low risk of GDM (of whom 15,127 [98.3%] were negative for GDM). However, this low-risk group contained 269 women who were confirmed positive for GDM later in pregnancy (30.4% of cases of GDM). Interestingly, our data suggests that these 269 women might not have been readily identified using risk-factor-based screening methods as approximately 38.7% were of normal pre-pregnancy BMI (<25 kg/m^2^). They had a 33.7% risk of having an LGA infant (Fig. 2).

**Fig. 3:**
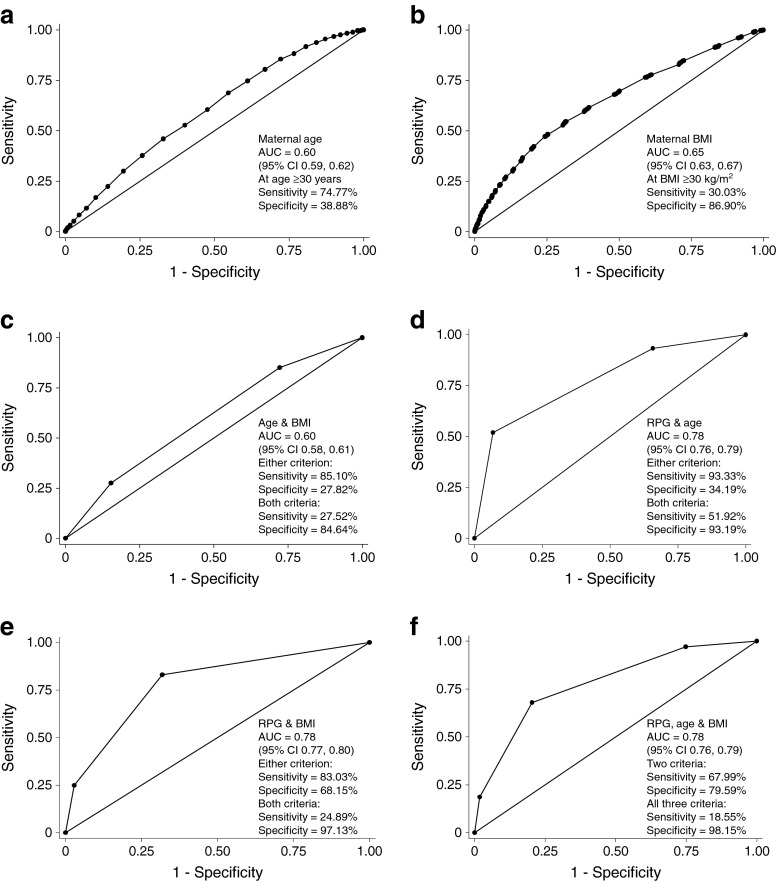
(**a**, **b**) ROC curves showing the use of maternal age at delivery (**a**) and pre-pregnancy BMI (**b**) for the prediction of GDM using the IADPSG criteria, as indicated in the graphs. (**c**–**f**) Combining the predictive value of RPG (≥7.5 mmol/l), maternal age (≥30 years) and maternal pre-pregnancy BMI (≥30 kg/m^2^)

The use of RPG at booking compared favourably with other screening strategies in current clinical use (Fig. 3a, b). For example, maternal pre-pregnancy BMI and maternal age were both inferior at predicting GDM using the IADPSG criteria (*n* = 1,181 positive diagnoses, *n* = 884 with RPG; BMI AUC 0.65, 95% CI 0.63, 0.67; age AUC 0.60, 95% CI 0.59, 0.62). Maternal age ≥30 years predicted IADPSG GDM with a sensitivity of 74.8% and a specificity of 38.9%. Maternal pre-pregnancy BMI ≥30 k/m^2^ predicted IADPSG GDM with a sensitivity of 0.30 and a specificity of 0.87. Combining the risk factors age and BMI with RPG did not improve the overall predictive ability compared with using RPG alone (Fig. 3c–f) when using thresholds of RPG ≥7.5 mmol/l, age ≥30 years and BMI ≥30 kg/m^2^. However, combining age and BMI (Fig. 3c), RPG and age (Fig. 3d) or RPG and BMI (Fig. 3e) gave an improvement in test sensitivity to 0.83–0.95, but at the cost of reducing the overall ROC AUC.

## Discussion

This retrospective study in 17,736 pregnant women demonstrates that RPG at antenatal booking has reasonable performance as a screening test for GDM and performs better overall than screening based upon established risk factors (maternal age and BMI). RPG may have a role in identifying women who are at low risk of GDM and who would be considered to be of relatively low priority for early diagnosis or screening. Conversely, RPG could be used to identify women at high risk of GDM who might benefit from earlier diagnosis or from more intensive lifestyle interventions in early pregnancy. However, women with a low RPG still can develop GDM. This data suggests that reliance on the RPG at booking alone, without universal testing in the second trimester, would miss around 30% of cases of GDM. The test sensitivity could be improved by combining RPG with risk-factor information for age or BMI, but with a reduction in the specificity and overall ROC AUC.

This study has several strengths. First, the large sample size and unselected nature of the population allows robust assessment of the validity of RPG in clinical practice. Second, the RPG measured at antenatal booking was followed by universal screening for GDM using a two-step GCT and 75 g OGTT protocol. However, this protocol is no longer recommended by international guidelines [4, 5] and, importantly, GDM has not been definitively excluded using an OGTT in all 17,736 women. This was a single centre study in a relatively mono-ethnic population with a low prevalence of GDM. The overall performance of any screening tests may vary in different populations with different prevalence rates for GDM [16]. Over 7,000 women did not have evidence of an RPG measurement. These women have been described elsewhere and are otherwise comparable with the general population giving no evidence of selection bias [17]. The blood was analysed in a single accredited laboratory or using point-of-care devices under established laboratory quality control protocols. However, during this period both capillary and venous testing was used for glucose quantification in our institution. This may introduce small differences between measured and actual glucose concentrations. We have no data about the timing of OGTT testing in relation to the RPG although most OGTTs were performed at around 28 weeks following a GCT at 24–28 weeks. Although we have detailed information on some maternal risk factors, such as obesity and age, we do not have consistent information about previous history of GDM or family history of type 2 diabetes.

The use of RPG has many advantages. First, it is inexpensive and can be performed during the antenatal booking visit with no special pre-test preparation. Second, the opportunity to use point-of-care analysis on a capillary sample allows the clinician to have prompt access to the results, facilitating early lifestyle intervention or confirmatory testing. This may be particularly beneficial in resource-poor or rural environments where women travel a great distance to appointments. However, the RPG result is affected by pre-testing conditions, such as food intake and exercise, and this gives a wide range of RPG values in both GDM-positive and GDM-negative populations.

Previous studies of the RPG have had conflicting results. van Leeuwen and colleagues prospectively assessed the validity of RPG vs OGTT in 322 pregnant women and found that the RPG at 24–28 weeks had an ROC AUC of 0.69 (95% CI 0.61, 0.78) [20]. The same authors also performed a systematic review assessing the validity of RPG in the diagnosis of GDM. Six papers met the entry criteria and included data from the Netherlands [20], China [21], Japan [22], UK [15], India [23] and Kuwait [24], with a broad range of prevalence rates for GDM. All the studies dealt with the performance of RPG in the second or third trimester except for one study by Maegawa and colleagues [22] who studied 749 pregnant women in Japan (2.9% had GDM). They found that the RPG and GCT had reasonable performance for detecting GDM in the first trimester. Some older studies indicated that GDM can be successfully diagnosed earlier in pregnancy [25–27], but the validity of using the WHO 2013 criteria outside the standard 24–28 week period has been questioned [28].

In our study, RPG with a sensitivity of 70% and specificity of 90% compares favourably with other approaches used for the screening of patients for GDM. A meta-analysis of the performance of the 50 g GCT, which included 13,564 women, showed that it had a sensitivity of 0.74 (95% CI 0.62, 0.87) and a specificity of 0.85 (95% CI 0.80, 0.91) for consecutive patients (not just those pre-selected based on risk factors) [29]. Other investigators have recommended a risk-factor-based approach. Göbl and colleagues designed a risk calculator based upon a woman’s history of previous GDM, glycosuria, family history of diabetes, age, pre-conception dyslipidaemia and ethnic origin [14]. The risk calculator in addition to a fasting plasma glucose concentration was able to predict GDM with a ROC AUC of 0.9. However, in clinical practice, collecting detailed risk-factor information can be challenging and prone to error and pre-conception lipid results are often unavailable. Interestingly, other reports from a different ethnic population did not support the use of fasting blood glucose or risk-factor-based screening. Dahanayaka and colleagues in Sri Lanka found that fasting blood glucose alone was a poor predictor of GDM and that a risk-factor-based approach was only able to identify around two-thirds of affected women [12].

This study indicates that RPG measurement could form part of a useful testing strategy to identify women in early pregnancy who are at risk of developing GDM, or to prioritise second trimester OGTT testing to those most at risk of GDM. Interestingly, although GDM is thought to develop after 20 weeks’ gestation in the majority of cases, this study shows that women in the first trimester can already be categorised biochemically according to their risk of later developing frank hyperglycaemia. In healthcare systems where universal biochemical screening with an OGTT is considered prohibitively expensive, RPG measurement at booking is likely to be a cost-effective and convenient way of identifying women who need to be prioritised for early lifestyle intervention and an OGTT in the second trimester.

## Electronic supplementary material

ESM Table 1 (PDF 54 kb)

## Abbreviations

ACOG: American Congress of Obstetricians and Gynecologists
FPG: Fasting plasma glucose
GDM: Gestational diabetes
GCT: Glucose challenge test
IADPSG: International Association of the Diabetes in Pregnancy Study Groups
LGA: Large for gestational age
NICE: National Institute for Health and Care Excellence
RPG: Random plasma glucose
USPSTF: US Preventative Services Task Force
