# Supplementary material for: Random plasma glucose in early pregnancy is a better predictor of gestational diabetes diagnosis than maternal obesity
Source: Diabetologia. 2015 Nov 20;59:445–52. doi: 10.1007/s00125-015-3811-5 (PMC4742503; doi:10.1007/s00125-015-3811-5)
Supplement: Supplementary file 1 — (PDF 54 kb) [file 125_2015_3811_MOESM1_ESM.pdf]

ESM Table 1: Current & recent criteria used for diagnosis of GDM based on the oral glucose tolerance test (OGTT)

| | IADPSG, WHO 2013 and ADA 2014 (11] | WHO 1999 | Modified WHO 1999 | NICE 2015 | American Congress of Obstetrics and Gynaecology (ACOG) |
|---|---|---|---|---|---|
| Diagnostic requirements | 1 abnormality on 75g OGTT | 1 abnormality on 75g OGTT | 1 abnormality on 75g OGTT | 1 abnormality on 75g OGTT | 2 abnormalities on 100g OGTT |
| Fasting plasma glucose mmol/l (mg/dl) | ≥5.1<br><br>(≥92) | ≥7.1<br><br>(≥128) | ≥6.1<br><br>(≥110) | ≥5.6<br><br>(≥101) | >5.3<br><br>(≥95) |
| OGTT 1hr glucose mmol/l (mg/dl) | ≥10.0<br><br>(≥180) | - | - | - | ≥10.0<br><br>(≥180) |
| OGTT 2hr glucose mmol/l (mg/dl) | ≥8.5<br><br>(≥153) | ≥7.8<br><br>(≥140) | ≥7.8<br><br>(≥140) | ≥7.8<br><br>(≥140) | ≥8.6<br><br>(≥154) |
| OGTT 3hr glucose mmol/l (mg/dl) | - | - | - | - | ≥7.8<br><br>(≥140) |
